# Editorial: The role of schools in adolescent mental health in low- and middle-income countries: considerations and future directions

**DOI:** 10.3389/fpsyt.2023.1307350

**Published:** 2023-10-23

**Authors:** Ruth Aston, Monika Raniti, Sachin Shinde

**Affiliations:** ^1^Faculty of Education, Assessment and Evaluation Research Centre, The University of Melbourne, Parkville, VIC, Australia; ^2^Centre for Adolescent Health, Murdoch Children's Research Institute, Melbourne, VIC, Australia; ^3^Department of Paediatrics, Melbourne Medical School, University of Melbourne, Melbourne, VIC, Australia; ^4^Department of Global Health and Population, School of Public Health, Harvard University, Boston, MA, United States

**Keywords:** whole-school approach, school-based mental health promotion, program, implementation, sustainability, low- and middle-income countries, adolescents, mental health interventions

Globally, adolescent mental health has gained prominence as a public health priority (1). Around one in five adolescents experience a mental health problem like anxiety each year and the COVID-19 pandemic has only intensified these concerns (2). Many common mental health problems such as depression typically have their first onset during the adolescent years when important physical, cognitive, behavioral, and social changes occur. Mental ill-health during these formative years can disrupt the attainment of developmental milestones such as those related to education and relationships, contributing to poorer physical and mental health later in life, increased health-care utilization, and threatening social and economic productivity. Conversely, mental wellbeing, social connection, and the acquisition of social-emotional skills during adolescence can contribute to more positive developmental trajectories. Thus, supporting mental health and preventing mental health problems during adolescence are imperative to reducing the individual and societal burden associated with mental ill-health and ensuring that future generations thrive.

School settings offer immense opportunities to support adolescents' mental health and learning and development (3). Most of the world's adolescents are enrolled in school and schools can play a vital role in the provision of health education and health services, particularly in low and middle-income countries (LMICs) where there may be challenges to delivery outside of schools. WHO and UNECSO's recent launch of the first global standards for health promoting schools (a whole-school systems approach to health and wellbeing) articulates the importance of the education sector in supporting students' health, highlighting that health and education are linked—education leads to better health and healthy students learn better. However, despite this global focus and that over 90 percent of the world's adolescents reside in LMICs, the majority of school mental health research has been conducted in high income countries (4–6); the evidence for how to support adolescent mental health in schools in LMICs is sorely lacking. This Research Topic of Frontiers in Psychiatry includes five articles focussing on LMICs which reinforce the value of schools in supporting adolescent mental health offering important learnings and directions for future research.

In Margaretha et al.'s school-based mental health global policy review, the authors investigated terminology in policy documents about school health programs and services published from 2000 to 2021. They found that the phrase “comprehensive school health” was used to refer to the *scope of program* where “comprehensive” referred to supporting mental health, preventing mental ill-health and promoting mental health; the *focus of program* where “comprehensive” referred to understanding mental health as connected to all dimensions of health including but not limited to nutrition, physical health, sexual and reproductive health; and the *approach of the program* where a “comprehensive” approach to supporting mental health is informed by a systems-approach, such as the global standards for Health Promoting Schools (7). The implication of their findings is that initiatives developed to ensure “comprehensive school health” could look very different, for instance “comprehensive” could refer to a program delivered across the intervention spectrum (e.g., preventing mental ill-health) and could also refer to a mental health program that supports mental health as well as physical activity and nutrition. A lack of shared terminology in relation to supporting adolescent mental health in schools poses challenges to developing the robust evidence base needed as the nature of interventions differ considerably making comparisons difficult and in fostering the inter-sectoral collaboration between health and education sectors.

Partap et al.'s cross-sectional study of six sub-Saharan African countries and Grande et al.'s meta-analysis of school-based programs for mental health problems among children and adolescents both highlight the need and benefits of school-based mental health support programs in LMICs. Examining programs in over twenty countries, the studies highlight that benefits such as increasing school attendance can be achieved through supporting mental health alongside other health outcomes for students and skills development. For example, Youth First was found to improve participants' inter- and intra-personal psychosocial skills such as problem-solving strategies and awareness of their own strengths (Leventhal et al.).

The sustainability of school-based mental health programs was found to be challenging. Shinde et al.'s exploratory case study of the SEHER trial identified the factors that explain the complex process of sustaining whole-school health promotion programs (8). The authors found that that school staff's understanding of the program philosophy, their capability to continue with the intervention activities, their motivation and attitude toward implementation, and the extent to which an enabling education policy environment and governance structure existed all influenced whether the SEHER program continued in school after initial implementation. Shinde et al. concluded that even if a program is designed as a whole-school initiative, and is effectively implemented, it does not necessarily follow that it will be embedded in the day-to-day operations of a school. Accordingly, planning for sustainability during initial implementation could increase the likelihood that whole-school approaches will be embedded in a school's “business as usual”.

It is noteworthy that all articles in this issue highlight that the effectiveness and sustainability of school-based mental health initiatives are underpinned by common prerequisite conditions; that school staff (leaders, teachers) and the wider school community have the capabilities, resources, motivation and will to continue to engage in such efforts as part of their day-to-day roles. Given that the authors collectively present a convincing view that in diverse settings school-based initiatives that support mental health can impact educational and health outcomes, it is imperative that future research focusses on the sustainability of school-based mental health initiatives. International research collaborations among education and health researchers could enable robust research to be conducted in LMICs, including developing a shared terminology and examining implementation and sustainability of school-based efforts from an interdisciplinary perspective. Generating evidence that uncovers how the necessary conditions for sustainability can be realized to support adolescents' mental health in LMICs could bolster the current global investment and focus on adolescent mental health for current and future generations.

## Author contributions

RA: Conceptualization, Writing—original draft, Writing—review and editing. MR: Conceptualization, Writing—review and editing, Writing—original draft. SS: Conceptualization, Writing—original draft, Writing—review and editing.
